# The Use of Non-Degradable Polymer (Polyetheretherketone) in Personalized Orthopedics—Review Article

**DOI:** 10.3390/polym17152158

**Published:** 2025-08-07

**Authors:** Gabriela Wielgus, Wojciech Kajzer, Anita Kajzer

**Affiliations:** Department of Biomaterials and Medical Devices Engineering, Faculty of Biomedical Engineering, Silesian University of Technology, Roosevelta 40 Street, 41-800 Zabrze, Poland; wojciech.kajzer@polsl.pl (W.K.); anita.kajzer@polsl.pl (A.K.)

**Keywords:** biomaterials, non-degradable polymers, additive technologies, personalized implants

## Abstract

Polyetheretherketone (PEEK) is a semi-crystalline thermoplastic polymer which, due to its very high mechanical properties and high chemical resistance, has found application in the automotive, aerospace, chemical, food and medical (biomedical engineering) industries. Owing to the use of additive technologies, particularly the Fused Filament Fabrication (FFF) method, this material is the most widely used plastic to produce skull reconstruction implants, parts of dental implants and orthopedic implants, including spinal, knee and hip implants. PEEK enables the creation of personalized implants, which not only have greater elasticity compared to implants made of metal alloys but also resemble the physical properties of the cortical layer of human bone in terms of their mechanical properties. Therefore, the aim of this article is to characterize polyether ether ketone as an alternative material used in the manufacturing of implants in orthopedics and dentistry.

## 1. Introduction

Biomedical engineering is a rapidly developing interdisciplinary field of science, which has played an important role in modern medicine since the end of the 20th century. Its continuous development is a response to the growing needs of modern medicine related to rehabilitation, the design of implants/medical instruments, and the improvement of diagnostic methods [1]. Continuous progress in diagnostics and medical technology has contributed to an increase in average human life expectancy. According to data from the Główny Urząd Statystyczny (eng. Central Statistical Office), it is projected that between 2002 and 2030, life expectancy for men in Poland will increase by approximately seven years, and for women by five years. Although this is a positive phenomenon, as it extends the biological age of humans, unfortunately, there has been an increase in the number of diseases and osteoarthritic conditions typical of an ageing population, including coxarthrosis (degeneration of the hip joint), gonarthrosis (degeneration of the knee joint), and spondylarthrosis (degenerative disease of the spine) [2,3,4,5].

After about the age of 25, the human body enters a period of slow ageing, resulting in a progressive deterioration of the functions of systems and organs. The most common phenomenon associated with ageing is the reduction in the repair and regenerative capacity of cells, which contributes to morphological degradation. Furthermore, after the age of fifty, a decrease in bone mineral density can be observed (especially in women), which most often leads to osteoporosis. Therefore, one of the main health problems in elderly people is joint degeneration, which results not only from the patient’s anatomical conditions, but also from the influence of the external environment [6].

**Figure 1:**
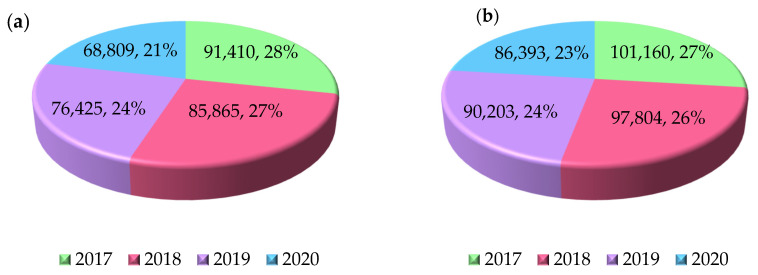
Number of people waiting for arthroplasty surgery in Poland [7]. (**a**) Hip joint. (**b**) Knee joint.

The proper development of effective treatment methods through joint replacement surgery is currently one of the key challenges of modern biomedical engineering, which aims at restoring the proper biomechanics of damaged joint structures, improve patients’ quality of life, and shorten recovery times. According to data from the Najwyższa Izba Kontroli (eng. Supreme Audit Office) from 2021, it can be observed that the number of patients waiting for hip or knee arthroplasty remains high. Despite the observed gradual reduction in waiting times, it is still not satisfactory. In 2020, the lowest number of patients waiting for surgery was recorded (Figure 1), which was a result of limited access to planned medical services due to the COVID-19 pandemic, which lasted in Poland until 1 July 2023 [7,8].

Based on data from the Narodowy Fundusz Zdrowia (eng. National Health Fund) from 2024, there were significant differences in waiting times for hip and knee replacement surgery depending on the region and specific medical facility.

For hip replacement surgery, the waiting time in the Mazowieckie province ranges from approximately 28 to 239 days, but in some medical facilities, the first available appointment is not until 2026 or even 2027. On the other hand, for knee replacement surgery, the Mazowieckie province offers a wide range of available dates, covering the period from 2024 to 2028. For comparison, in the Wielkopolska province, the waiting time for surgery exceeds 12 months, and in some medical facilities, the first available appointments are scheduled for 2030 [9].

In summary, osteoarthritis is a significant health and social problem, especially in the context of an ageing population. Progressive degradation of joint structures leads to chronic pain, limited range of motion, and deterioration in patients’ quality of life. In many cases, the only effective treatment is joint replacement surgery [7,8,9].

## 2. Characteristics of Implants in Orthopedics

Orthopedic implants are classified as long-term implants, which are an important element of modern biomedical engineering. They play a significant role in the treatment of degenerative diseases and congenital defects occurring in the osteoarticular system [10,11]. They are most often made of biomaterials: metal, ceramic or polymer, which are characterized by diverse chemical composition, structure, as well as physicochemical and mechanical properties [12,13,14]. These properties should guarantee bone stabilization and support for surrounding tissues during the remodeling and regeneration process. It should be emphasized that there is currently no material that meets all the requirements for orthopedic implants. Therefore, the suitability and application of a specific material should be considered individually in terms of its future use [10,11].

### 2.1. Materials Used in the Production of Orthopedic Implants

**Figure 2:**
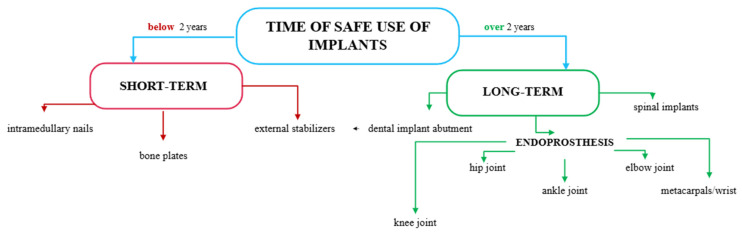
Classification of implants according to the duration of use and biomaterial selection [own elaboration].

Metal alloys are most used in the production of orthopedic implants, as they provide the appropriate mechanical and physicochemical properties and meet the requirements for the safe use of a given biomaterial in the body—Figure 2.

For this purpose, biomaterials are used for short-term implants, which should not remain in the body for more than two years (e.g., austenitic steel [15]), and long-term implants, such as cobalt-based alloys [16]. They are characterized by significantly greater resistance to pitting and crevice corrosion in body fluids than Cr-Ni-Mo steel, which means that these alloys can be used to manufacture long-term implants with a service life of more than two years [17].

Cobalt alloys are characterized by a very high Young’s modulus—Table 1, which significantly exceeds that of the cortical layer of human bone (17,700 MPa). This leads to stress compensation in the implant–bone system. As a result, bone density gradually decreases, ultimately leading to loosening, displacement or damage/fracture of the implant. In such cases, revision arthroplasty was often necessary. To limit undesirable phenomena, titanium alloys (e.g., Ti6Al4V, Ti6Al7Nb, Ti13Nb13Zr) [18,19,20] began to be used in the production of orthopedic implants as an alternative to the above-mentioned biomaterials. Titanium alloys are characterized by adequate lightness and high strength, which ensures good mechanical adaptation to the properties of the cortical bone layer, while supporting the regeneration process of the surrounding tissues [17,21,22]. Despite the widespread use of Ti6Al4V alloy in the production of orthopedic implants, it contains elements (aluminium and vanadium) that can cause adverse reactions in the body. Vanadium is classified as a toxic element and can cause inflammation in the human body and even metallosis. On the other hand, excessive aluminium aggregation in tissues may be associated with the onset of Alzheimer’s disease, among other things. Therefore, new titanium alloys have been developed for use in orthopedic implants—Ti6Al7Nb and Ti13Nb13Zr, which are intended to replace toxic elements and improve corrosion resistance [23,24,25,26,27].

Polymer materials have become an alternative to metal biomaterials and are widely used in modern medicine and biomedical engineering—a detailed description of these materials can be found in Section 3.

### 2.2. Manufacturing Technologies Used in the Production of Orthopedic Implants

To produce a suitable orthopedic implant, after selecting the biomaterial (usually in the form of a semi-finished product), it is very important to choose the right manufacturing technology. This is crucial for the functionality of the implant, its integration with bone tissue, and for minimizing the risk of post-operative complications. Traditional manufacturing methods include rolling, forging, turning, milling, drilling, and threading. In the case of implants intended for short-term use, the final stage of processing is grinding and electromechanical polishing. The aim of these processes is to improve the tribological and physicochemical properties of the surface of the medical device [21,35]. However, in contrast to short-term implants, long-term implants are not polished to maintain the appropriate surface roughness, which promotes proper integration of the implant with the bone tissue, thus supporting the osseointegration process.

Additive technologies, which revolutionized industry and medicine in the late 1990s, have become an alternative to traditional implant manufacturing methods. Additive technologies have enabled the creation of personalized implants for specific conditions, which has significantly improved the effectiveness of patient treatment [36,37,38,39]. In particular, the production of orthopedic implants uses metal alloy powder sintering techniques, using a laser or electron beam, which are classified as PBF (Powder Bed Fusion) technologies. The most widely used techniques include SLM (Selective Laser Melting), DMLS (Direct Metal Laser Sintering), and EBM (Electron Beam Melting). The most important advantages of powder technologies include the high density of solid parts, the high degree of metal alloy melting, and the ability to precisely reproduce complex geometries. However, it should be noted that despite the high melting of the material, unmelted powder particles may remain in the structure of the manufactured product. For this reason, sandblasting is an essential stage of surface treatment after the manufacturing process, the purpose of which is to remove unbound material grains. This allows us to produce products with an appropriately porous structure that supports the osseointegration process [40,41,42,43,44,45]. Furthermore, these technologies enable the creation of structurally complex implants with a gradient structure (variable density from solid parts to porous structures produced in a single process).

However, in addition to additive technologies based on sintering metal alloy powders, there is also a steady development of additive technologies using polymer filaments, which has opened new possibilities in the design and manufacture of orthopedic implants. The most widely used printing technology in this field is FFF (Fused Filament Fabrication). The manufacturing process involves the layered application of thermoplastic material (filament), which after the print head reaches the appropriate temperature (depending on the type of material used), is plasticized to a semi-liquid state and then precisely applied to the working platform [39]. These technologies enable the precise reproduction of complex anatomical structures, which are used, among other things, in skull bone reconstructions, spinal implants and endoprostheses, e.g., hip or knee joints [46,47,48,49,50].

Solutions using additive technologies offer fast implementation of even the most complex projects and cost reductions compared to traditional material processing methods. However, it should be remembered that regardless of the implant manufacturing technology used, each product undergoes a detailed analysis to verify whether the medical device can be implanted in the patient’s body.

## 3. Polymers in Medicine

Contemporary medicine is constantly evolving towards the use of advanced biomaterials that improve patients’ quality of life and the effectiveness of their treatment. An important group among these materials are plastics, whose use in medicine began by accident. During World War II, pilots who suffered plane crashes sustained numerous injuries, including those resulting from contact with fragments of disintegrating aircraft cockpits. However, doctors observed that fragments of cockpit cover made of polymethyl methacrylate (PMMA) did not cause additional allergic reactions or other adverse responses from the body. This observation led to PMMA being used as the first synthetic biomaterial, which in turn sparked the dynamic development of polymers in medical applications [11,51].

### 3.1. Classification of Polymers for Medical Applications

Polymers are a group of organic compounds whose macromolecules are composed of many smaller particles called monomers. The particles can be arranged in an orderly and repetitive manner, allowing for the formation of long chains with diverse physicochemical properties. Depending on the number of types of monomers in the chain, polymers are divided into homopolymers containing only one type of monomer and copolymers consisting of at least two different particles. Plastics used in modern medicine can be divided into two basic groups: natural polymers and synthetic polymers. Each of these groups includes both biodegradable and non-biodegradable materials. Natural polymers have found application in tissue engineering, where they are valued for their biocompatibility and biodegradability. Natural polymers include, among others: collagen, chitin, and chitosan [45,52].

Non-biodegradable synthetic polymers are used in medicine to manufacture elements that stabilize bone fractures. Despite their favorable mechanical properties and high biocompatibility, some implants are damaged. One of the most common problems is insufficient osteoconduction, often resulting from low implant surface roughness, which leads to loosening and the formation of microcracks at the implant–bone interface. This group of synthetic polymers includes polyethylene, polyetheretherketone, and the polymethyl methacrylate. Biodegradable synthetic polymers, on the other hand, are mainly used in regenerative medicine, where they serve as temporary elements supporting the tissue regeneration process [51].

### 3.2. Characteristics of PEEK in Orthopedic Implants

Polyetheretherketone (PEEK) is part of a broad group of thermostable ketone polymers called polyaryletherketones (PAEKs). The polymers in this family are characterized by a diverse chemical composition, resulting from different proportions of both ether and ketone groups in the molecular chain. Of all the representatives of the PAEK group, it is PEEK that is most used in industrial and medical applications. It was first synthesized in 1977 and its commercial use began in 1981. The material is classified as a homopolymer with a linear structure. Its characteristic feature is its high molecular weight—a single polymer chain is composed of hundreds of repeating monomer units and reaches an average molecular weight in the range of 80,000–120,000 g/mol [53,54].

Polyetheretherketone is used for the manufacture of orthopedic implants, and in particular knee and hip joint replacements, as well as implants for spinal stabilization. Due to its favorable mechanical and chemical properties, it is also used in dentistry, particularly as a material for prosthetic reconstruction and implant components [55,56,57,58].

#### 3.2.1. Properties of Polyetheretherketone

**Figure 3:**
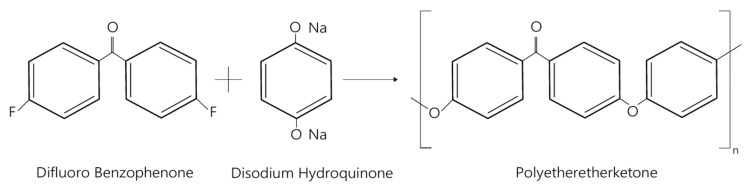
Structure of polyetheretherketone [own elaboration].

The synthesis of PEEK is most often based on the reaction of difluorobenzophenone with disodium hydroquinone by polycondensation (Figure 3). The length of the resulting polymer chain, determined by the reaction conditions, directly influences material properties such as glass transition temperature (Tg) or mechanical strength, allowing for their controlled modification. Tg is defined as the limiting temperature below which the polymer chains are in an amorphous state. In this temperature range, the macromolecular segments have too low a thermal energy to move freely relative to each other. As a result, the material exhibits mechanical properties characteristic of the solid state. For PEEK, the glass transition temperature is around 143 °C, which demonstrates its high thermal stability and the possibility of using under elevated temperature conditions without losing structural integrity [53].

PEEK combines very good mechanical properties—(Table 2) and tribological properties, as well as dimensional stability. An important feature of PEEK is its resistance to high operating temperatures, which allows for long-term operation in demanding environmental conditions. In addition, this polymer is characterized by high resistance to ultraviolet radiation, X-rays, and has a very low VOC (volatile organic compound) content [22,53,59].

Literature data indicate [51,60] that PEEK can provide an alternative to the most widely used crystallization process titanium alloys in orthopedics and trauma treatment. One key advantage is its lower Young’s modulus compared to metal alloys. This allows for the stiffness of the implant to better match the cortical layer of the bone [50,51,53,59].

#### 3.2.2. Crystallinity of PEEK

The molecular chain of polyetheretherketone is characterized by a long, irregular structure in which the molecules are not static, but are subject to vibration and rotation under the influence of thermal energy and deformation. The rigidity of the molecule is due to the presence of aromatic benzene rings in its backbone, but it has the freedom to rotate axially around ether (-O-) and ketone (-CO-) bonds. During slow cooling from the molten state, the molecular chains organize themselves into ordered crystalline structures, forming folds and forming crystals embedded in the amorphous phase, resulting in a semi-crystalline microstructure. PEEK exhibits a two-phase character, consisting of a crystalline and an amorphous phase, the proportions of which depend on the heat treatment parameters, especially the cooling rate. Increasing the cooling rate reduces the crystallinity, leading to a material with an almost completely amorphous structure [53].

#### 3.2.3. The Technological Process of Creating a Personalized PEEK Implant

**Figure 4:**
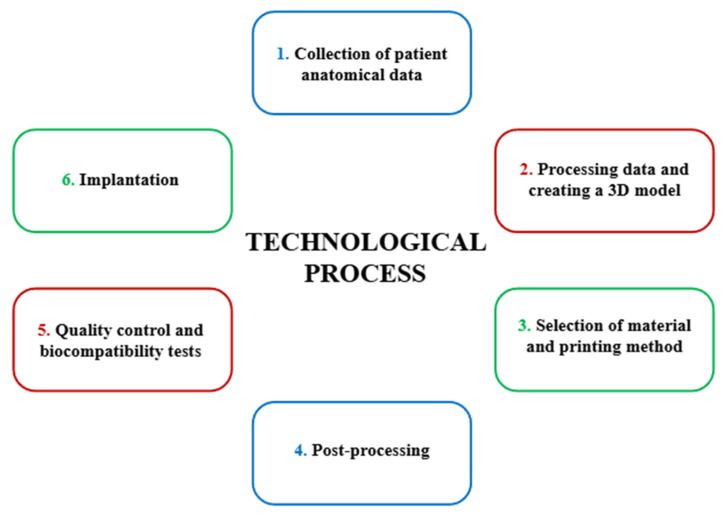
Technological process of creating personalized implants [own elaboration].

A schematic diagram of the technological process involved in the creation of a personalized implant is shown in Figure 4.

The first stage preceding the production of a personalized orthopedic implant is the collection of the patient’s anatomical data, which is obtained based on computed tomography (CT) or magnetic resonance imaging (MRI). The data obtained is saved in DICOM (Digital Imaging and Communications in Medicine) format, which is then processed to create a digital 3D model, for example, of the knee joint. Using specialized CAD (Computer-Aided Design) software, the image is segmented and then the DICOM data is converted into a spatial digital mesh, saved in STL (Standard Tessellation Language) format. Based on the model prepared in this way, an implant is designed, whose geometry is adjusted and modified to meet the anatomical and biomechanical requirements of the patient. The third stage involves the selection of appropriate material and manufacturing technology. In the case of personalized PEEK implants, the filament used in FFF technology is most used. After printing the implant, an extremely important step is post-processing, which involves removing support structures (necessary during 3D printing) and heat treatment to obtain a semi-crystalline structure that significantly improves the strength properties of the material. In addition, the surface of the implant is modified to promote better osseointegration, for example, these issues are described in detail in Section 3.2.4. The final stage before implantation is quality control and assessment of the biocompatibility of the finished product. Each implant must meet the requirements of the Medical Device Regulation (MDR) and undergo appropriate biocompatibility testing (e.g., in vitro tests). After a positive result, it is possible to perform the implantation of the medical device in the human or animal body [43,61,62,63,64].

#### 3.2.4. Additive Technologies Using PEEK for Personalized Orthopedic Implants

**Figure 5:**
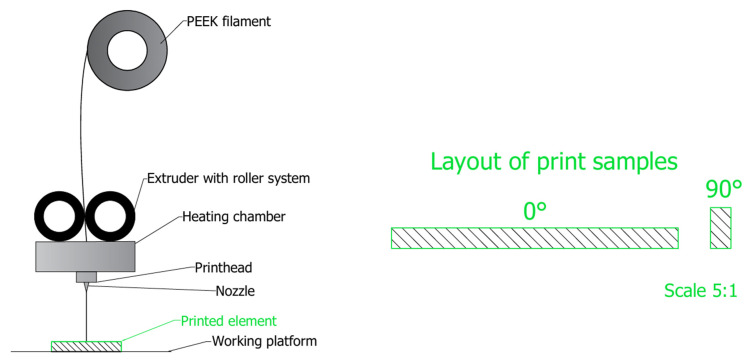
Schematic of the FFF printing process with the arrangement of the samples on the work platform [own elaboration].

For the manufacture of personalized orthopedic implants, described in Section 2.2, the PEEK filament in FFF technology is most used. This material is characterized by a semi-crystalline structure, the control of which during the printing process is a fundamental factor influencing the quality and mechanical properties of the final product. In order to ensure proper interlayer adhesion and minimize defects in the structure of the printed implant, it is recommended to conduct the printing process under high temperature conditions—the work platform should be kept at a temperature of about 100 °C, the extruder at a temperature of about 400 °C, and the work chamber at a temperature of about 120 °C. In addition, it is recommended that the specimens are arranged in a 0°/90° directional pattern with a ±45° printing orientation (Figure 5), which promotes a homogeneous structure and improves the mechanical properties of the implant, in particular, increasing the bending strength [61,65,66,67,68].

Specimens printed in 0° orientation showed a flexural strength of 122.53 MPa, while specimens printed in 90° orientation reached 156.84 MPa. Zhen et al. also highlighted the effect of interlayer bond quality on mechanical strength [61]. Samples printed in horizontal (0°) orientation have weaker interlayer bonding, which is due to lower adhesion between layers compared to stronger fiber bonds within a single layer.

To analyze in detail the influence of printing parameters such as print orientation, fill angle and extrusion rate of the filament, the authors conducted tensile and bending tests. The results of these tests are presented in Table 3, which showed the extreme values of the mechanical properties obtained at different printing parameter settings [61].

Analysis of the results allows for the optimization of the PEEK implant manufacturing process to obtain products with the best possible performance properties. An extrusion rate that is too low (0.8×) results in insufficient filling and poor interlayer bonding, which reduces tensile (31.78 MPa) and flexural (56.48 MPa) strengths. On the other hand, too high an extrusion speed (1.2×) deteriorates print quality due to the formation of defects such as air pores and gaps between layers, resulting in a decrease in tensile strength. The most favorable properties were obtained at the standard extrusion speed (1.0×), where the tensile strength reached 69.35 MPa. Samples printed horizontally and vertically show similar tensile strengths, but in the case of bending, the vertical orientation significantly improves the mechanical performance, reaching 156.84 MPa [61].

However, to maintain adequate mechanical properties for orthopedic implants, it is recommended to print with a 100% fill rate and a layer height in the range of 0.15–0.20 mm. On the other hand, to increase the mechanical properties of the material (for orthopedic implants), it is best to heat-treat the material at a temperature of approximately 150 °C after printing to achieve a semi-crystalline material structure [66,67,68,69,70,71]. This is also confirmed by the results of a study presented by the authors [61], who showed that it is the temperature of the heat treatment that plays a key role in modifying the mechanical properties, while its duration does not significantly affect the change in mechanical properties. Detailed test results showing the dependence of mechanical properties on heat treatment parameters are summarized in Table 4.

Compared to the results shown in Table 3, the tensile strength of the heat-treated samples increased from 70.84 to 74.24 MPa, while the flexural strength increased from 157.37 to 167.13 MPa [61]. Furthermore, a crystallinity test was carried out to observe the differences in strength tests, which are shown in Table 5.

Differential scanning calorimetry (DSC) was used to determine the quantitative ratio of crystalline to amorphous phases. The crystallinity of the polyetheretherketone increases with increasing temperature and heat treatment time, which corresponds to an improvement in mechanical properties such as tensile and flexural strength. In the initial state, the crystallinity of the samples was 16.10 percent, while a marked increase in this parameter is observed after heat treatment at 150 °C, reaching 18.03 percent after just 0.5 h and increasing to 23.77 percent after 2 h. Therefore, heat treatment is an important final processing step, recommended to increase crystallinity and improve the strength parameters of the material [61].

Also based on observations made using scanning electron microscopy (SEM) after mechanical testing, the authors of the publication [67] noted a significant effect of heat treatment on the mechanical properties of the material. Due to the heat treatment, a more efficient remelting of the layers, stronger interlayer bonds, and a reduced number of voids were observed compared to samples in the uncured state [67].

In its initial state, polyetheretherketone is characterized by hydrophobic properties, so that it does not exhibit adequate bioactivity (it does not promote osteointegration, which limits its effectiveness in applications requiring direct connection of the implant to bone tissue). To improve its hydrophilic properties and the osteointegration process, surface modifications such as the application of coatings with, for example, hydroxyapatite, which increase the surface energy of the polymer, are often used. This type of modification promotes cell adhesion, cell proliferation, and bone tissue growth around the implant. Furthermore, the hydrophilic properties of PEEK not only influence the integration with bone tissue, but also the interaction with microorganisms [56,72,73,74]. Composites containing polyetheretherketone with the addition of multi-walled carbon nanotubes (CNTs) or bioactive glass are also created to improve osseointegration. The use of such composites allows for the modification of the implant surface, which leads to a change in the surface energy of PEEK and thus promotes better integration with bone tissue. Carbon nanotubes are characterized by excellent mechanical properties, significantly exceeding the parameters of ceramics and metal alloys used. Their tensile strength ranges from 11 000 to 52 000 MPa, their flexural strength is 14,200 ± 8000 MPa, and their Young’s modulus reaches values from 32,000 to 1,470,000 MPa. It is also worth noting that carbon nanotubes are extremely light, with a density ranging from 1.3 to 2 g/cm^3^. Bioactive glasses (BGs), on the other hand, are biodegradable ceramic materials used in the human body as components of implants that rebuild damaged bone tissue. They consist mainly of CaO and P_2_O_5_, and a layer of hydroxyapatite forms on their surface, which, after dissolving in the tissue environment, enables the formation of strong bonds between the implant and the bone [75].

The most used methods of physical modification of PEEK surfaces include plasma treatment, laser treatment, UV radiation, and Ion Beam Etching (IBE). Plasma treatment allows for the introduction of functional groups using gases such as oxygen, nitrogen, argon, water vapor and ammonia, which leads to increased hydrophilicity and surface roughness, promoting cell adhesion [76,77]. Ion etching can be used to increase surface roughness, which will further contribute to the surface energy of the material. IBE (Ion Beam Etching), also known as ion grinding, was developed in the 1970s as a technique for the physical etching of surfaces. The process involves bombarding the target material with accelerated inert gas ions (e.g., argon). This procedure also aims to improve the wettability and adhesion of potential functional layers. In addition, to achieve high adhesion, amorphous layers of hydrogenated silicon carbonyl nitride have been produced on the surface, providing an effective intermediate layer that promotes permanent bonding to the material in the initial state [78,79].

In vitro studies have shown that such modifications significantly improve the adhesion, spreading, proliferation, and early differentiation of osteoblasts, which in turn may promote faster osseointegration and induce bone tissue maturation in the immediate vicinity of the implant [76].

Biofilm formation on the surface of PEEK is comparable to or even less than with zirconium oxide or titanium [80]. In addition, an important advantage of the above plastic is its high resistance to chemical and environmental degradation, thus avoiding complications characteristic of some metallic implants, such as metallosis [22,51,56,60,65,81,82].

Intensive work is still underway to improve the antibacterial properties of implants, as postoperative infections are among the most common complications in bone tissue engineering and orthopedics. One of the most used approaches is the use of antibiotics loaded into thermosensitive hydrogels based on poly (lactic-co-glycolic acid) (PLGA) and polyethylene glycol (PEG)-PLGA copolymers. The authors of publication [83] used vancomycin loaded in a hydrogel for a porous PEEK scaffold with high mechanical strength. In vivo studies conducted on a rat model demonstrated the effective antibacterial activity of this system. In addition to antibiotics, antimicrobial peptides (AMPs) are attracting increasing interest due to their good water solubility, thermal stability, tissue non-toxicity, and broad spectrum of antibacterial activity [84].

## 4. Conclusions

The development of modern engineering materials and additive technologies has initiated new possibilities in the design and manufacture of personalized orthopedic implants. Among the materials that have revolutionized the approach to treating osteoarthritis, polyetheretherketone has a special place. It offers an attractive alternative to traditional metal alloys such as titanium, especially in the context of implants individually tailored to the patient’s condition. A comparison with alternative materials used for orthopedic implants is presented in Table 6.

Both the physicochemical and mechanical properties of PEEK show a significant dependence on the parameters of the 3D printing process. The orientation of the samples with respect to the printing direction has a significant impact on their strength properties. Studies have shown that samples produced in a 90° orientation (perpendicular to the work platform) have higher mechanical strength compared to samples printed at 0°. The polymer has a resistance to high temperatures and a Young’s modulus similar to that of the cortical layer of bone, which is a significant advantage over traditionally used metallic biomaterials. To increase the roughness and improve the adhesion of PEEK, it is possible to use ion etching, which leads to an increase in the surface energy of the material. This allows modified PEEK to find wider application in tissue engineering and in the production of personalized medical implants, such as knee or hip implants [61,66,67,68,69,70,71,72,73].

## Figures and Tables

**Table 1:** Mechanical properties of selected metal biomaterials [15,16,18,19,20,28,29,30,31,32,33,34].

Material	R_m_ [MPa]	R_p0,2_ [MPa]	A [%]	E [MPa]	ρ [g/cm^3^]
Cr-Ni-Mo	860	690	10	193,000	8.0
CoCrMo	1000	700	12	227,000	8.4
Ti6Al4V	860	795	10	112,000	4.4
Ti6Al7Nb	900	800	10	110,000	4.5
Ti13Nb13Zr	973	836	10	79,000	4.9
Cortical bone	200	*	1.4	17,700	1.6–2.0

* For cortical bone, compressive strength is given—R_c_ = 145 MPa. R_m_—tensile strength, R_p0,2_—yield strength, A—elongation after fracture, E—Young’s modulus, ρ—density.

**Table 2:** Mechanical properties of PEEK [14,47,48].

R_m_ [MPa]	R_p0,2_ [MPa]	A [%]	E [MPa]	ρ [g/cm^3^]
116	116	15	4044	1.3

**Table 3:** Strength test results of samples produced using different printing parameters [61].

Strength Tests		Tensile [MPa]	Bending [MPa]
**Orientation of Test** **Samples**	0°	67.14	122.53
	90°	66.97	156.84
**Angle Fills**	±10°	69.35	144.16
	±30° lub ±20°	56.65	122.53
**Extrusion Speed**	0.8×	31.78	56.48
	1× 1.2×	69.35	160.88

**Table 4:** Effect of heat treatment and time on strength properties [61].

Strength Tests		Tensile [MPa]	Bending [MPa]
**Temperature**	150 °C	70.84	157.37
	300°	74.24	167.13
**Time**	30 min	74.24	167.13
	2 h	77.26	172.98

**Table 5:** Polyetheretherketone crystallinity test results [61].

Parameters		
Temperature [°C]	Time [h]	Crystallinity [%]
150	0.5	18.03
	1	22.98
	2	23.77

**Table 6:** Alternative materials used for orthopedic implants [85,86,87,88].

Material	Young’s Modulus [MPa]	Fatigue Resistance [MPa]	Osseointegration	Clinical Performance
Ti6Al4V	112,000	510	very good	orthopedic implants
PEEK	4044	121	requires surface modification to improve osseointegration	hip and knee implants
UHMWPE	790	*		hip and knee implants
Ceramics (ZrO_2_)	200,000	*	it has anticatarrhal properties and supports the remodeling process	hip joint socket

* No numerical values.

## Data Availability

Not applicable.

## Abbreviations

The following abbreviations are used in this manuscript:

PEEK	Polyetheretherketone
FFF	Fused Filament Fabrication
PBF	Powder Bed Fusion
SLM	Selective Laser Melting
DMLS	Direct Metal Laser Sintering
EBM	Electron Beam Melting
PMMA	Polymethylmethacrylate
PAEKs	Polyaryletherketones
CT	Computed Tomography
MRI	Magnetic Resonance Imaging
DICOM	Digital Imaging and Communications in Medicine
CAD	Computer-Aided Design
STL	Standard Tessellation Language
MDR	Medical Device Regulation
VOCs	Volatile Organic Compounds
SEM	Scanning Electron Microscopy
IBE	Ion Beam Etching
CNTs	Carbon Nanotubes
DSC	Differential Scanning Calorimetry
BGs	Bioactive Glasses
PLGA	Poly(lactic-co-glycolic acid)
PEG	Polyethylene Glycol
